# Radiant Heat for the Relief of Pain

**Published:** 1898-03-19

**Authors:** 


### RADIANT HEAT FOR THE RELIEF OF PAIN.

The importance of heat as a means of relieving pain is well known not only to the medical profession but to all who have had anything to do with the management of patients suffering from painful ailments. The difficulty is in its application. Dr. Hedley, writing in *Treatment*, enters into the different modes by which the application of heat can be effected. The method generally adopted has been to apply it by means of a medium—something hot, such as hot water or hot air—but, as Dr. Hedley points out, if exclusively radiant heat be employed no such media are necessary. The air through which the radiation travels may be as cold as ice, but the body upon which the heat rays fall becomes warm nevertheless. As soon as the temperature of a body rises above that of the surrounding objects radiation of heat takes place, and the heat rays may be detected and measured, but the intense radiation required for the purpose now under discussion hardly takes place until the heat-producing source has attained such a temperature that luminous rays are also given off. After considering the various available sources of incandescent heat Dr. Hedley comes to the conclusion that far the best is to be found in the electric light. He then goes on to consider the sort of apparatus which would be required for the therapeutic application of radiant heat, and describes one which was exhibited at a recent meeting of the Balneological and Climatological Society.

This possibly may be the germ of something useful, but at present there seem difficulties in the way. Of course, everyone knows that exposure to the radiant heat of a glowing fire will relieve neuralgia and other like forms of pain, but there are certain inconveniences attached to this method, especially in regard to its irritating effect upon the skin, which will probably always limit its utility.

